# Gait performance in cerebral small vessel disease: associations with cognitive and oculomotor function and exploratory mediation analyses

**DOI:** 10.3389/fnagi.2026.1935861

**Published:** 2026-09-16

**Authors:** Chen-Xin Shen, Xiao-Ying Zhao, Shi-Yao Wang, Yan-Wei Miao, Zhan-Hua Liang, Lin Liu, Bing-Wei Zhang

**Affiliations:** 1 Department of Neurology, First Affiliated Hospital of Dalian Medical University, Dalian, Liaoning, China; 2 Department of Neurology, Central Hospital of Dalian University of Technology, Dalian, Liaoning, China; 3 Department of Radiology, First Affiliated Hospital of Dalian Medical University, Dalian, Liaoning, China; 4 Department of Psychology, Dalian Medical University, Dalian, Liaoning, China

**Keywords:** cerebral small vessel disease, executive function, gait impairment, mediation analysis, oculomotor function

## Abstract

**Background:**

Gait impairment in cerebral small vessel disease (CSVD) is heterogeneous and may not be fully explained by conventional structural MRI markers. We investigated the associations of neuroimaging burden, cognitive function, and oculomotor function with gait performance in CSVD and explored potential indirect associations between CSVD burden and gait impairment.

**Methods:**

We included 65 individuals with CSVD and 35 control participants. All participants underwent quantitative gait assessment, standardized neuropsychological evaluation, and oculomotor recording. Patients with and without gait disorder were compared after adjustment for the total CSVD load score. Correlation analyses assessed associations among neuroimaging markers, cognitive and oculomotor measures, and gait parameters. Exploratory mediation analyses examined cognitive and oculomotor measures as potential statistical indirect pathways between total CSVD load and gait performance.

**Results:**

Compared with control participants, patients with CSVD had poorer gait, cognitive, and oculomotor performance (*P* < 0.05). Despite comparable total CSVD load, patients with gait disorder showed worse executive function and visuospatial ability, prolonged saccade latency, reduced fixation stability, and lower anti-saccade accuracy (FDR-adjusted *P* < 0.05). Total CSVD load and perivascular spaces (PVS) burden were associated with cognitive, gait, and oculomotor measures. In the exploratory mediation analyses, Trail Making Test-B performance showed a significant indirect association between total CSVD load and gait impairment, with no significant residual direct association. Trail Making Test-A performance, fixation stability, and saccade latency, also showed significant indirect association, while the corresponding direct associations remained significant.

**Conclusions:**

Executive and oculomotor dysfunction were associated with gait impairment in CSVD and may partly account for the association between total CSVD burden and gait performance. These exploratory findings require confirmation in larger longitudinal studies.

## 1 Introduction

Cerebral small vessel disease (CSVD) is frequently encountered in later life and has become an important source of both vascular cognitive decline and walking difficulty in elderly individuals (Wardlaw et al., 2019). When gait is affected in this condition, patients are more likely to fall, lose independence in daily living, and experience unfavorable long-term outcomes, including death (Zhou et al., 2020). These mobility problems also interfere with everyday functioning and perceived health status (Bower et al., 2019). However, the relationship between brain imaging abnormalities and gait performance is not straightforward. In clinical practice, patients with a similar amount of white matter hyperintensities or lacunar infarcts may walk very differently (Xie et al., 2025). Some remain able to ambulate with little apparent limitation, whereas others develop obvious slowing, instability, or other motor deficits (Sharma et al., 2023; Wu et al., 2024). Such variability suggests that the amount of visible small-vessel damage on conventional imaging may not be sufficient to explain gait impairment. Other mechanisms, possibly involving cognition, eye movement control, or broader brain-network disruption, may help explain why similar radiological damage leads to different motor outcomes.

Gait depends on more than the execution of leg movements. To walk safely and adapt to the environment, individuals must continuously use sensory feedback together with cognitive control (Berlot et al., 2021; Cicirelli et al., 2022). Executive function, attention, and visuospatial processes are therefore closely tied to gait regulation (Osoba et al., 2019). Earlier research has tested whether particular CSVD lesions, most often white matter hyperintensities, relate to gait speed, balance, or other gait features, yet the results have varied across studies (Pinter et al., 2017; Loos et al., 2018). The total CSVD load score also appears to explain only a small proportion of interindividual variation in gait performance. This implies that widespread small-vessel damage may contribute to walking problems, but additional mechanisms are likely to be involved. Interpretation of prior findings is further complicated by insufficient control of disease severity between groups, which may have influenced comparisons of motor function (Jayakody et al., 2021; Zhao et al., 2022).

Recording eye movements may provide useful information about brain-circuit function through a simple and non-invasive assessment. Oculomotor control relies on a broad network involving the pre-frontal cortex, basal ganglia, brainstem, and cerebellum; these regions are also important for cognition and coordinated movement (Alizadeh and Van Opstal, 2022). In Parkinson's disease and other neurodegenerative disorders, disturbances in saccades, smooth pursuit, or fixation often coexist with cognitive impairment and freezing of gait (Wu et al., 2020). These observations raise the possibility that oculomotor parameters could serve as markers of circuit vulnerability in CSVD.

Accordingly, we investigated the relationships among CSVD burden, cognitive and oculomotor function, and gait impairment. Gait, cognition, and eye movements were assessed using wearable sensors, a standardized neuropsychological battery, and eye-tracking tasks, respectively. Patients with and without gait impairment were compared after adjustment for total CSVD load and other structural MRI markers to reduce confounding by disease severity. Mediation analysis was then used to examine cognitive and oculomotor measures as potential statistical indirect pathways between CSVD burden and gait performance. To our knowledge, this is one of the first studies to evaluate cognition and oculomotor function together in relation to gait impairment in CSVD.

## 2 Materials and methods

### 2.1 Participants

A total of 75 patients with CSVD were initially recruited. 10 patients who did not complete all required assessments were excluded, leaving 65 patients in the final analytical cohort. In addition, 35 control participants were included. Patients were consecutively recruited from the Neurology Department of the First Affiliated Hospital of Dalian Medical University between November 2022 and August 2023. The hospital's Ethics Committee approved the study protocol [No. PJ-KS-2023-04(X)], and written informed consent was obtained from each participant. CSVD was diagnosed according to the 2020 Chinese guidelines on the diagnosis and management of CSVD. Patients were required to have at least one vascular risk factor, one or more MRI markers of CSVD (e.g., white matter hyperintensities of presumed vascular origin, recent small subcortical infarcts, cerebral microbleeds, lacunes, together with enlarged perivascular spaces), and at least one clinically documented manifestation potentially related to CSVD, including cognitive, gait, emotional, urinary, or bowel symptoms.

Eligible patients in the CSVD group were 50–80 years old, right-handed, scored ≤ three points on the modified Rankin scale, and had the ability to complete brain MRI, gait, neuropsychological, and oculomotor assessments reliably. Patients were excluded if they had an acute symptomatic cerebral infarction at the time of assessment; stroke attributed to large-artery atherosclerosis, cardioembolism, cryptogenic or other uncommon causes; epilepsy, multiple sclerosis, Parkinson's disease, Alzheimer's disease, or another neurological disorder affecting cognition, eye movements, or gait; a major psychiatric disorder; a history of traumatic brain injury, neurosurgery, or intracerebral hemorrhage; severe systemic disease; spinal, hip, or knee disorders independently affecting gait; MRI contraindications; or severe sensory, visual, cognitive, language, writing, motor, or oculomotor impairment that precluded reliable completion of the study tasks.

Based on their Timed Up and Go Test (TUG) completion time, participants were classified into the CSVD-GD group (TUG ≥ 11.5 s) and the CSVD-NGD group (TUG < 11.5 s). The classification was based solely on gait performance and was independent of cognitive and oculomotor results. Total CSVD load and individual MRI markers were compared between the two patient groups rather than assumed to be matched.

### 2.2 Data collection

#### 2.2.1 Assessment of demographic and clinical characteristics

Demographic and clinical data were obtained through structured interviews and review of medical records. Collected variables encompassed age, sex, body mass index (BMI), completed years of formal education, smoking and alcohol use, hypertension, diabetes, and atrial fibrillation.

#### 2.2.2 Acquisition and processing of neuroimaging data

Neuroimaging data were obtained and processed after the clinical evaluation. Imaging used a standardized multi-parametric protocol on a 3.0-Tesla magnetic resonance imaging (MRI) scanner that included T1-weighted, fluid-attenuated inversion recovery (FLAIR), T2-weighted, susceptibility-weighted imaging (SWI), and magnetic resonance angiography (MRA). White matter hyperintensities (WMH) were rated separately in the periventricular and deep white matter using the Fazekas scale, with scores ranging from zero to three. Enlarged perivascular spaces (PVS) were scored with Potter's scale, and cortical volume loss was assessed with the global cortical atrophy (GCA) scale. The number of cerebral microbleeds (CMBs) and lacunes was determined based on international guidelines. A total CSVD load score, ranging from zero to four, was calculated as the sum of the individual item scores. Two neuroradiologists, blinded to all clinical and functional data, independently rated the MRI scans. Disagreements were resolved by a third neuroradiologist.

#### 2.2.3 Gait assessment

Clinical gait and balance were assessed using the Tinetti Balance and Gait Scale, and functional mobility was evaluated using the TUG test. The TUG test was performed three times, and the mean of the final two trials was used in the analysis. Spatiotemporal gait parameters were recorded with the Ruiping V2.0 wearable sensor system (Hunan Gyenno Medical Technology Co., Ltd., Hunan, China). Recorded parameters included step length, stride length, cadence, gait speed, stance and swing phase duration, stride length variability, and turning time. Five-times sit-to-stand performance was also recorded. Detailed sensor placement and assessment procedures are provided in Supplementary methods.

#### 2.2.4 Evaluation of neuropsychological and behavioral functions

Global cognition was assessed using the Montreal Cognitive Assessment (MoCA) and the Mini-Mental State Examination (MMSE). Processing speed and set-shifting were measured using Parts A and B of the Trail Making Test, respectively. Visuospatial ability was evaluated with the Judgment of Line Orientation (JLO) scale and the Clock Drawing Test (CDT). Language function was assessed with the Boston Naming Test (BNT), and auditory-verbal short-term memory with the Digit Span Test (DST). Emotional states were measured with the Hamilton Anxiety Rating Scale (HAMA) and the Hamilton Depression Rating Scale (HAMD). Dependence in daily activities was measured with the Activities of Daily Living (ADL) scale, and autonomic symptoms were assessed using the Overactive Bladder Symptom Score (OABSS).

#### 2.2.5 Assessment of oculomotor function

Oculomotor performance was recorded with the EyeKnow eye-tracking platform (Beijing CAS-Ruiyi Information Technology Co., Ltd, Beijing, China). Following five-point calibration, each participant undertook four oculomotor tasks: overlap saccade, anti-saccade, lateral gaze, and smooth pursuit. The principal measures used in the analyses were overlap saccade latency, anti-saccade accuracy, lateral gaze deviation and accuracy, and horizontal smooth pursuit deviation, whereas additional oculomotor measures were analyzed as exploratory variables. Invalid individual trials were automatically excluded, and oculomotor measures were calculated from the remaining valid trials; all participants had sufficient valid trials for analysis. Detailed task protocols, variable definitions, and data-processing procedures are provided in Supplementary methods.

#### 2.2.6 Data preprocessing and quality control

Data were screened for completeness, accuracy, and potential outliers before statistical analysis. Patients who did not complete all required assessments (*n* = 10) were excluded before the final analytical cohort was established. Complete data for the primary analysis variables were available for all included participants (65 patients with CSVD and 35 control participants), and no missing value imputation was required. Potential outliers were verified against the original records and retained when confirmed to be valid. No outlier removal or winsorization was performed. All variables were analyzed in their original units without normalization or standardization.

### 2.3 Statistical analysis

Continuous variables are presented as mean ± standard deviation or median with interquartile range, as appropriate, and categorical variables as frequencies and percentages. The Shapiro-Wilk test was used to assess normality and homogeneity of variance was evaluated using Levene's test. Comparisons between the CSVD and HC groups were performed using independent-samples *t*-tests or Mann–Whitney *U*-tests according to data distribution. Comparisons among HC, CSVD-NGD, and CSVD-GD groups were performed using one-way ANOVA or the Kruskal–Wallis test, followed by pairwise comparisons when the overall test was significant. Categorical variables were compared using chi-square or Fisher's exact tests, as appropriate. Correlations were assessed using Pearson's correlation coefficient for normally distributed variables and Spearman's rank correlation coefficient otherwise. *P*-values from the correlation analyses and pairwise group comparisons were adjusted using the Benjamini–Hochberg false discovery rate (FDR) procedure. The adjusted *P*-values are reported in the relevant tables.

Mediation analyses were performed to estimate the total, direct, and indirect associations of total CSVD load with gait performance through cognitive and oculomotor mediators. Candidate mediators were selected based on their relevance to the proposed pathways and their observed associations with total CSVD load and gait measures. No prospective sample size calculation was performed for the indirect effects. Therefore, all mediation analyses were considered exploratory and hypothesis-generating rather than confirmatory. Models were adjusted for age, years of education, body mass index, hypertension, and history of stroke. Indirect effects were estimated using 5,000 bias-corrected bootstrap resamples and were considered statistically significant when the 95% confidence interval excluded zero. For significant indirect effects, the proportion mediated was calculated as the indirect effect divided by the total effect. Because the mediators were evaluated after the initial association analyses, these models were considered exploratory.

To examine directional robustness, a reverse-model sensitivity analysis was performed by switching TMT-B completion time and TUG time in the model with total CSVD load as the exposure. The bootstrap 95% confidence interval for the reverse indirect effect included zero. Given the cross-sectional design, these results do not establish causality or temporal precedence.

All analyses were conducted using JASP version 0.18.3 (University of Amsterdam, Amsterdam, Netherlands). A two-sided *P* < 0.05 was considered statistically significant unless FDR correction was specified; for FDR-corrected analyses, an adjusted *P* < 0.05 was considered statistically significant.

## 3 Results

### 3.1 Participant characteristics and neuroimaging burden

The analytical cohort comprised 65 patients with CSVD and 35 control participants. The groups did not differ in age, sex distribution, BMI, or years of education (all *P* > 0.05). Hypertension and a history of stroke were more prevalent in the CSVD group than in the control group (70.8% vs. 31.4% and 40.0% vs. 2.9%, respectively; both *P* < 0.001). The prevalence of diabetes mellitus and atrial fibrillation did not differ significantly between the groups (both *P* > 0.05; Table 1).

**Table 1:** Baseline demographic, vascular, and MRI-derived characteristics of the study participants.

Variable	CSVD group (*n* = 65)	Control group (*n* = 35)	*P*
Basic characteristics			
Age (years)	67.46 ± 6.43	64.94 ± 5.94	0.058
Women, *n* (%)	33 (50.8)	23 (65.7)	0.151
Body mass index (kg/m^2^)	24.72 ± 2.65	24.84 ± 2.52	0.832
Years of education	9.89 ± 3.58	10.12 ± 2.59	0.793
Cerebrovascular risk profile			
Hypertension, *n* (%)	46 (70.8)	11 (31.4)	< 0.001^***^
History of stroke, *n* (%)	26 (40.0)	1 (2.9)	< 0.001^***^
Diabetes mellitus, *n* (%)	18 (27.7)	9 (25.7)	0.832
Atrial fibrillation, *n* (%)	2 (3.1)	1 (2.9)	1.000
MRI markers of CSVD severity			
Total CSVD load score	3 (3, 4)	2 (1, 3)	< 0.001^***^
Fazekas-PV WMHs	3 (2, 3)	1 (1, 2)	< 0.001^***^
Fazekas-DWM WMHs	2 (1, 3)	0 (0, 1)	< 0.001^***^
PVS-Potter score	3 (2, 4)	0 (0, 1)	< 0.001^***^
CMB anatomical score	2 (0, 4)	0 (0, 0)	< 0.001^***^
Global cortical atrophy score	1 (0, 1)	0 (0, 1)	0.159

Values are expressed as mean ± standard deviation, number with percentage, or median with interquartile range, as appropriate. CSVD, cerebral small vessel disease; PV, periventricular; WMH, white matter hyperintensity; DWM, deep white matter; PVS, perivascular space; CMB, cerebral +microbleed; MRI, magnetic resonance imaging. ^***^P < 0.001.

Compared with controls, CSVD patients had higher Fazekas scores for periventricular and deep WMH, greater PVS burden on the Potter scale, higher cerebral microbleed (CMB) anatomical score, and higher total CSVD load scores (all *P* < 0.001). Global cortical atrophy ratings, by contrast, did not differ between the two groups (*P* > 0.05, Table 1).

### 3.2 Group differences in gait, cognitive, oculomotor, neuropsychiatric, and daily living measures

Compared with the control group, the CSVD group had longer TUG times, lower Tinetti assessment scores, slower gait speed, shorter step lengths, and greater stride length variability. The CSVD group also had lower MMSE, MoCA, and CDT scores and longer completion times on Trail making tests A and B. For the oculomotor measures, CSVD patients showed longer overlap saccadic latency, lower anti-saccade accuracy, and lower lateral gaze accuracy than the control group. They also had higher HAMA, HAMD, and ADL scores. All between-group differences remained statistically significant after correction for multiple comparisons within the prespecified functional domains (all FDR-adjusted *P* ≤ 0.003; Table 2).

**Table 2:** Comparison of multidimensional functional performance between CSVD patients and healthy control participants.

Functional category	Assessment variable	CSVD group (*n* = 65)	Control group (*n* = 35)	FDR-adjusted *P*
Gait and balance	Timed up and go, s	11.26 (10.18, 15.52)	8.68 (7.75, 9.79)	0.001^**^
	Tinetti assessment score	26 (22, 28)	28 (28, 28)	0.001^**^
	Gait speed, m/s	0.77 ± 0.21	1.03 ± 0.18	0.001^**^
	Step length, cm	45.07 ± 11.41	57.97 ± 7.82	0.001^**^
	Variability of stride length, %	6.42 (4.57, 9.63)	4.25 (2.92, 6.40)	0.001^**^
Cognitive function	MMSE	24 (21, 27)	28 (28, 30)	< 0.001^***^
	MoCA	20 (16, 23)	26 (25, 27)	< 0.001^***^
	Trail making test A, s	75 (50, 133)	50 (38, 67)	< 0.001^***^
	Trail making test B, s	191 (152, 287)	125 (100, 155)	< 0.001^***^
	Clock drawing test score	9 (6, 10)	10 (10, 10)	< 0.001^***^
Oculomotor function	Overlap saccade latency, ms	370.93 ± 80.69	311.45 ± 49.72	0.001^**^
	Anti-saccade accuracy, %	18.34 (0.00, 46.59)	59.38 (44.27, 85.71)	0.003^**^
	Lateral gaze accuracy, %	83.53 (73.74, 89.99)	91.69 (89.57, 92.72)	0.001^**^
Neuropsychiatric and daily function	HAMA	10 (7, 16)	6 (4, 9)	0.001^**^
	HAMD	9 (5, 13)	5 (3, 8)	< 0.001^***^
	ADL	20 (20, 24)	20 (20, 20)	< 0.001^***^

Values are expressed as mean ± standard deviation for variables with normal distribution and as median with interquartile range for skewed variables. *P* values were adjusted using the Benjamini–Hochberg false discovery rate procedure separately within each pre-specified functional domain. ^**^P < 0.01 and ^***^P < 0.001. CSVD, cerebral small vessel disease; MMSE, Mini-Mental State Examination; MoCA, Montreal Cognitive Assessment; HAMA, Hamilton Anxiety Scale; HAMD, Hamilton Depression Scale; ADL, Activities of Daily Living.

### 3.3 Cognitive and oculomotor characteristics of CSVD patients with and without gait disorder

To compare clinical, neuroimaging, cognitive, and oculomotor characteristics according to gait disorder status, CSVD patients were stratified into those with gait disorder (CSVD–GD; *n* = 30) and those without gait disorder (CSVD–NGD; *n* = 35). No statistically significant subgroup differences were observed in age, sex distribution, vascular risk profiles, CMB anatomical score, and total CSVD load (all FDR-adjusted *P* > 0.05).

As expected from the subgroup classification, the CSVD–GD group had longer TUG times and slower gait speed than the CSVD–NGD group (both FDR-adjusted *P* < 0.001). The CSVD–GD group also had lower MMSE, MoCA, CDT and JLO scores and longer completion times on TMT-A and B (all FDR-adjusted *P* ≤ 0.004). BNT and DST scores did not signigicantly differ between the subgroups (FDR-adjusted *P* = 0.223 and 0.200, respectively).

For the oculomotor measures, the CSV–DGD group with gait disturbance had more lateral gaze deviations, longer overlap saccade latency, a lower anti-saccade accuracy, and more horizontal smooth pursuit deviations than the CSVD–NGD group (all FDR-adjusted *P* ≤ 0.049; Table 3). The FDR correction included all assessed measures within each corresponding domain, including measures not displayed in Table 3. Results for the remaining measures are provided in Supplementary Table 1.

**Table 3:** Clinical, imaging, cognitive, and oculomotor characteristics of CSVD patients stratified by gait disorder status.

Category	Variable	CSVD with gait disorder (*n* = 30)	CSVD without gait disorder (*n* = 35)	FDR-adjusted *P*
Neuroimaging	Total CSVD load score	3.5 (3, 4)	3 (3, 4)	0.140
	Anatomical score for cerebral microbleeds	3 (0, 5.5)	1 (0, 3)	0.095
Gait performance	Timed up and go, s	15.55 (13.36, 17.80)	10.21 (9.03, 10.78)	< 0.001^***^
	Gait speed, m/s	0.64 ± 0.18	0.90 ± 0.15	< 0.001^***^
Cognition	MMSE	22 (19, 24)	26 (24, 28)	< 0.001^***^
	MoCA	17 (12, 18)	23 (20, 25)	< 0.001^***^
	Trail making test A, s	102 (67, 191)	63 (48, 79)	0.004^**^
	Trail making test B, s	249 (191, 377)	164 (144, 200)	< 0.001^***^
	Clock drawing test score	7 (4, 9)	9 (8, 10)	0.004^**^
	Judgment of line orientation score	16 (11, 19)	21 (17, 23)	< 0.001^***^
	Boston naming test score	25 (21, 27)	25 (23, 27)	0.223
	Digit span test score	14 (12, 17)	15 (13, 18)	0.200
Oculomotor function	Lateral gaze deviation count (>2°)	29.50 (15.50, 62.75)	21.00 (10.50, 30.00)	0.009^**^
	Overlap saccade latency (ms)	395.14 ± 77.96	349.58 ± 78.02	0.049^*^
	Anti-saccade accuracy (%)	10.10 (0.00, 27.70)	33.33 (7.42, 60.00)	0.009^**^
	Horizontal smooth pursuit deviation count (>4°)	51.00 (41.75, 72.50)	35.00 (20.00, 52.25)	0.006^**^

Values are shown as mean ± standard deviation or median with interquartile range, as appropriate. CSVD, cerebral small vessel disease; The two CSVD subgroups were comparable in age, sex distribution, and vascular risk factors (all adjusted *P* > 0.05). *P* values are Benjamini–Hochberg false discovery rate (FDR)-adjusted separately within each pre-specified functional domain. ^*^P < 0.05, ^**^P < 0.01, ^***^P < 0.001. CSVD, cerebral small vessel disease; CSV–DGD, cerebral small vessel disease with gait disorder; CSVD–NGD, cerebral small vessel disease without gait disorder; CMB, cerebral microbleed; MMSE, Mini-Mental State Examination; MoCA, Montreal Cognitive Assessment.

### 3.4 Correlation among neuroimaging, gait, cognitive, and oculomotor measures

Figure 1 summarizes the correlations among neuroimaging markers, oculomotor, cognitive, and gait measures that remained statistically significant after FDR correction. High total CSVD load scores were correlated with longer TUG times (*r* = 0.34, FDR-adjusted *P* < 0.001), lower Tinetti assessment scores (*r* = −0.44, FDR-adjusted *P* < 0.001), poorer MMSE (*r* = −0.34, FDR-adjusted *P* < 0.001) and MoCA scores (*r* = −0.35, FDR-adjusted *P* < 0.001), longer TMT-A (*r* = 0.39, FDR-adjusted *P* < 0.001) and TMT-B (*r* = 0.42, FDR-adjusted *P* < 0.001) completion times, and longer overlap saccade latency (*r* = 0.33, FDR-adjusted *P* = 0.001). Among individual imaging markers, higher PVS Potter scores were correlated with longer TUG times, lower Tinetti assessment scores, lower MMSE and MoCA scores, longer TMT-A and TMT-B completion times, longer overlap saccade latency, and higher lateral gaze deviation counts. Significant correlations were also observed between gait, cognitive, and oculomotor measures (Figure 1).

**Figure 1:**
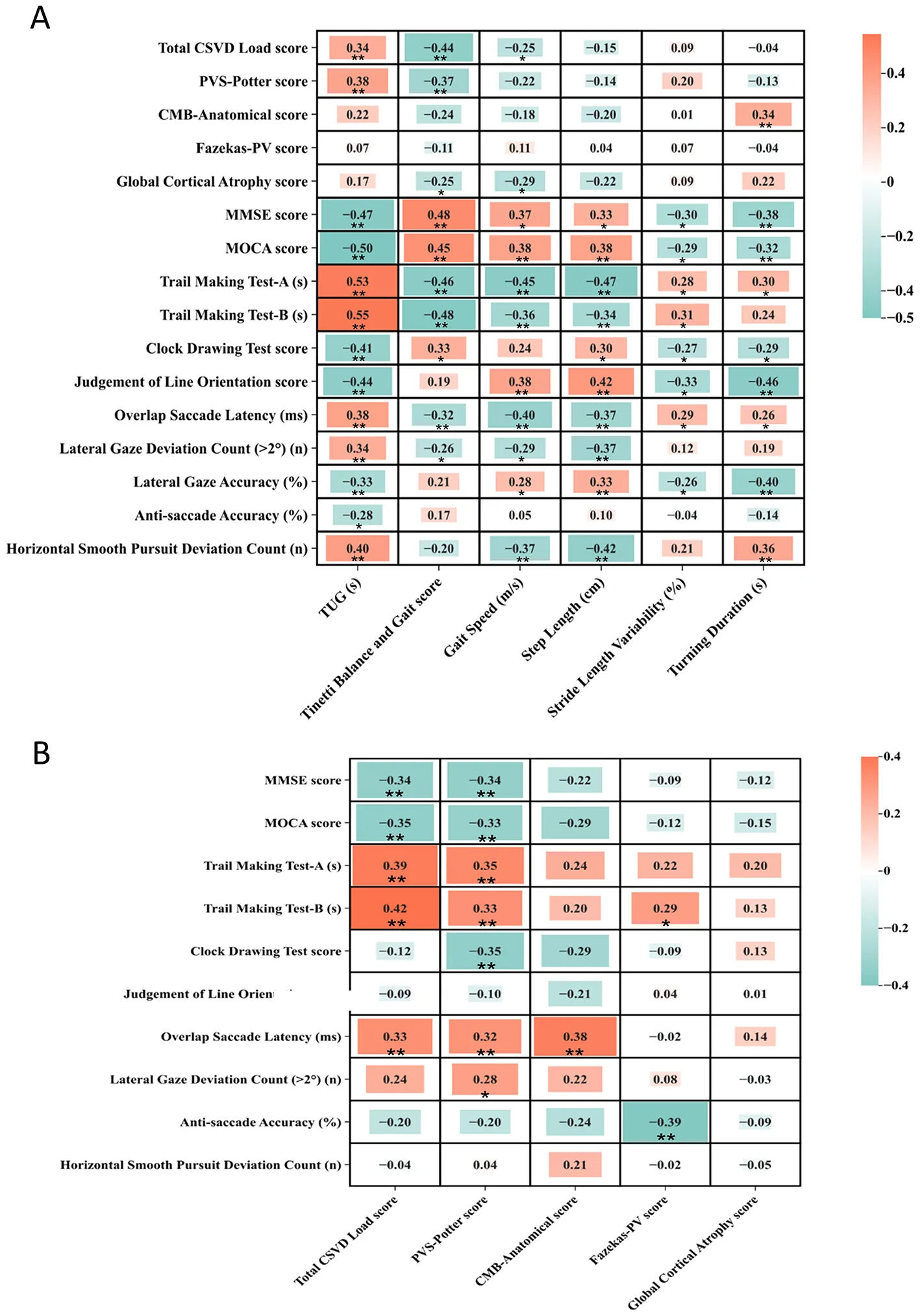
Heatmap of correlations among gait, neuroimaging, cognitive, and oculomotor measures. **(A)** Correlation analysis between gait parameters and neuroimaging markers, cognitive scores and oculomotor metrics. **(B)** Correlation analysis between neuroimaging markers and cognitive and oculomotor metrics. Cell colors represent the direction and magnitude of the Pearson correlation coefficient (*r*). Statistical significance was assessed using a two-tailed test against zero correlation and *P* values adjusted for multiple comparisons using the Benjamini–Hochberg false discovery rate procedure. *P < 0.05; **P < 0.01.

### 3.5 Mediation analyses of associations between CSVD burden and gait performance

Covariate-adjusted mediation analyses were performed to estimate the indirect associations of CSVD-related neuroimaging markers with TUG completion time through cognitive and oculomotor measures. A statistically significant indirect effect of total CSVD load on TUG completion time through TMT-B completion time was observed (indirect effect = 0.81, 95% *CI* 0.21–1.76; Figure 2A). The corresponding direct effect was not statistically significant (*P* > 0.05). TMT-A completion time was also associated with a significant indirect effect (indirect effect = 0.56, 95% *CI* 0.12–1.27), with an estimated proportion mediated of 32.6% (Figure 2B). Similarly, overlap saccade latency showed a significant indirect effect in the association between total CSVD load and TUG completion time (indirect effect = 0.33, 95% *CI*: 0.02–0.92), with an estimated proportion mediated of 19.9% (Figure 2C).

**Figure 2:**
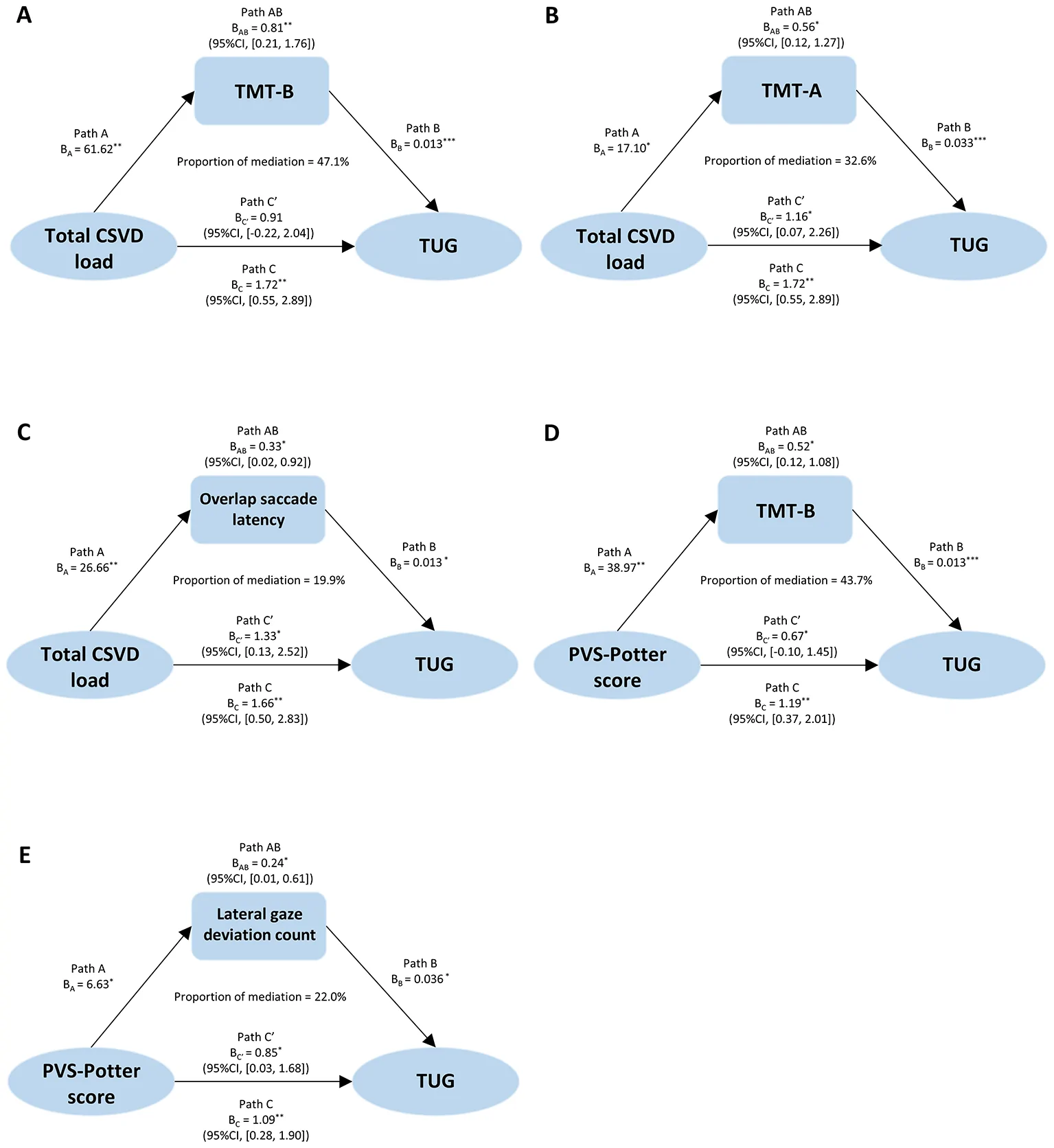
Mediation models of the associations between CSVD-related neuroimaging burden and TUG completion time through cognitive and oculomotor measures. **(A–C)** Show models of the associations between total CSVD load and TUG completion time through TMT-B completion time, TMT-A completion time, and overlap saccade latency, respectively. **(D, E)** Show models of the associations between PVS severity and TUG completion time through TMT-B completion time and lateral gaze deviation count, respectively. Path diagrams present unstandardized regression coefficients **(B)** with 95% bootstrap confidence intervals. Standardized indirect effects (β) were 0.15 for Model 1 **(A)**, 0.10 for Model 2 **(B)**, 0.06 for Model 3 **(C)**, 0.12 for Model 4 **(D)**, and 0.05 for Model 5 **(E)**. *P < 0.05; **P < 0.01; ***P < 0.001. CSVD, cerebral small vessel disease; TUG, Timed Up and Go; TMT, Trail Making Test; PVS, perivascular spaces.

For PVS severity, a statistically significant indirect effect on TUG completion time through TMT-B completion time was observed (indirect effect = 0.52, 95% *CI* 0.12–1.08; Figure 2D). Lateral gaze deviation count also showed a significant indirect effect in the association between PVS severity and TUG completion time (indirect effect = 0.24, 95% *CI*: 0.01–0.61), with an estimated proportion mediated of 22.0% (Figure 2E).

In a reverse-direction sensitivity analysis, TUG completion time was specified as the mediator and TMT-B completion time as the outcome, with total CSVD load retained as the exposure. The reverse indirect effect was not statistically significant (indirect effect = 0.13, 95% *CI*: −0.07–0.38), as the bootstrap 95% *CI* included zero.

## 4 Discussion

In clinical practice, gait impairment in patients with CSVD does not always correspond closely to the apparent severity of imaging abnormalities. Patients with a relatively similar neuroimaging burden may nevertheless exhibit substantially different gait performance. To investigate this clinical heterogeneity, we combined gait analysis, cognitive testing, and eye-movement assessment, and compared CSVD patients with and without gait disorder after adjusting the two groups for total CSVD load. The results showed that the gait disorder group had poorer executive function, visuospatial performance, and oculomotor control. Among the imaging markers examined, PVS burden assessed using the Potter scale showed the most consistent associations with gait and cognitive measures. Exploratory mediation analyses suggested that executive function, processing speed, and selected oculomotor measures may partly account for the association between total CSVD load and TUG performance. To our knowledge, few studies have examined cognitive and oculomotor function together in patients with CSVD while accounting for overall disease burden. Our findings suggest that gait impairment in CSVD is related not only to visible brain lesions but also to cognitive-executive and oculomotor dysfunction.

These results suggest that executive dysfunction may help explain why CSVD patients with greater imaging burden tend to show poorer gait performance. In other words, cognition appears to be more than a parallel clinical feature; it may partly account for the association between CSVD burden and gait impairment. This conclusion aligns with the cognitive mediation model proposed by Cai et al. (2021), which suggested that changes in structural network efficiency may be associated with gait through cognitive impairment. The abnormalities observed in selected oculomotor measures are also consistent with previous evidence that oculomotor performance can capture subtle circuit-level dysfunction in CSVD (Pinkhardt et al., 2014). However, our findings appear to differ from those of studies involving genetically defined CSVD, such as CADASIL, where the relationship between cognition and gait has been reported to be weaker (Finsterwalder et al., 2019). This difference may be related to the characteristics of the study populations. CADASIL cohorts are usually younger and may be less affected by age-related systemic problems. By contrast, patients with sporadic CSVD in the present study were older and may therefore have been more likely to have factors such as reduced muscle strength, reduced proprioception, visual impairment, or subclinical vestibular dysfunction. Under these conditions, gait may already be less automatic and more dependent on attentional and executive control. Any additional CSVD-related cognitive decline may be more likely to be associated with overt gait impairment. Thus, the prominent indirect association involving cognition in our sample may reflect the combined influence of disease pathology and reduced sensorimotor reserve with aging.

Among the imaging markers examined, PVS burden showed the most consistent associations with both executive and gait impairment. Accumulating evidence suggests that enlarged PVS may be associated with CSVD-related disability, rather than merely representing an incidental imaging finding of vascular injury (Lin et al., 2022; Elias-Mas et al., 2024; Yang et al., 2025). One possible explanation for the observed associations is that PVS enlargement may reflect impaired perivascular fluid transport or glymphatic clearance (Gouveia-Freitas and Bastos-Leite, 2021; Yu et al., 2022; Ai et al., 2025). Experimental and human studies have associated perivascular pathology and enlarged PVS with glymphatic-related abnormalities, altered perivascular diffusion, and vascular injury (Hein and Che Mohd Nassir, 2026a,b,c). Because glymphatic clearance was not directly assessed in this study, the observed associations should be interpreted as exploratory. Altered perivascular function could potentially be associated with neuroinflammation, disruption of neural homeostasis, and broader network dysfunction affecting both cognition and gait, but these mechanisms cannot be confirmed by the present data.

When the mediation results are considered together with the correlation networks, several possible explanations emerge. CSVD-related gait impairment may not follow a single pathway. In some patients, diffuse white matter and subcortical injury may affect fronto-subcortical circuits involved in executive control, attention, and visuospatial processing. These cognitive functions are needed for anticipatory postural control and rapid adjustment of gait in changing environments (Kim et al., 2014; Wilson et al., 2019; Zhou et al., 2020). Because frontal and subcortical regions are also involved in oculomotor control, damage within these circuits may contribute to the parallel abnormalities in cognition, eye movements, and gait observed in our study. Another pathway may involve perivascular dysfunction. A higher PVS burden may reflect altered perivascular homeostasis and has been associated with glymphatic-related abnormalities and neuroinflammatory processes (Wardlaw et al., 2020; Chong et al., 2025; Ineichen et al., 2022; Fu et al., 2025). However, PVS are non-specific imaging markers, and the present study did not directly assess glymphatic clearance or neuroinflammation. Therefore, the relationship between PVS burden and these biological processes remains speculative. If perivascular abnormalities are accompanied by disruption of broader sensorimotor integration networks, visuomotor coordination may become less efficient, making walking less stable and potentially more dependent on compensatory cognitive control (Vassar and Rose, 2014; Deravet et al., 2018). This may be particularly relevant in older patients, whose sensorimotor reserve is already reduced. This framework suggests that gait disorder in CSVD is unlikely to be a simple motor outcome. It may instead be associated with different combinations of cognitive, oculomotor, and sensorimotor network dysfunction. Such a view may help explain why patients with similar conventional imaging burden can show very different gait profiles. It also raises the possibility that oculomotor measures could help detect brain network dysfunction and identify patients at higher risk of later mobility decline, although their predictive value requires longitudinal validation.

These findings may have clinical implications. Oculomotor measures, particularly saccade latency and fixation stability, may complement routine assessment in identifying CSVD patients with gait impairment, including those whose gait symptoms are not yet clinically apparent. However, whether these measures can predict subsequent gait decline remains unknown. The present results also suggest possible rehabilitation targets, such as executive function training, gaze stabilization exercises, and visuomotor feedback training. Whether these interventions can prevent or slow gait decline in CSVD remains to be tested. Moreover, basal ganglia PVS burden may provide additional information about gait impairment beyond conventional measures of CSVD lesion burden, although its value for risk stratification requires prospective validation.

Several limitations should be considered. First, the cross-sectional design precludes conclusions about causality or temporal sequence. Longitudinal studies are needed to determine whether cognitive and oculomotor measures predict subsequent mobility decline. Second, the single-center recruitment and modest sample size may limit the generalizability and precision of the findings. No prospective sample size calculation was performed for the indirect effects, and several candidate mediators were examined, raising concerns about model stability and false-positive findings. The mediation results should therefore be considered exploratory and require validation in larger, independent, multi-center cohorts. Third, neuroimaging markers were assessed using semiquantitative visual rating scales and lesion counts, which may introduce measurement imprecision and do not fully characterize structural brain injury. We did not assess regional WMH volume, tract-specific damage, diffusion abnormalities, or structural connectivity, which may explain additional variation in gait performance. Future studies using automated lesion segmentation, regional lesion mapping, diffusion MRI, and structural connectivity analyses could provide a more precise assessment of lesion distribution and network disruption related to gait impairment. Fourth, although the mediation models were adjusted for several demographic and vascular factors, other potential determinants of gait, including frailty, sarcopenia, visual or peripheral sensory impairment, musculoskeletal disorders, and vestibular dysfunction, were not comprehensively assessed. Therefore, residual confounding cannot be excluded. Finally, although FDR correction was applied to group comparisons and correlation analyses, the exploratory mediation models were not adjusted for multiple testing, and false-positive findings remain possible. Future confirmatory studies should prespecify primary mediators and apply appropriate multiplicity correction or independent validation.

In summary, executive dysfunction and oculomotor abnormalities were associated with gait impairment in patients with CSVD and were identified as significant statistical indirect pathways between MRI-defined CSVD burden and gait performance. Basal ganglia PVS burden was also associated with cognitive, oculomotor, and gait measures. These findings suggest that cognitive and oculomotor assessments may complement conventional MRI markers in characterizing gait impairment in CSVD. However, the cross-sectional design does not establish causality, temporal sequence, or predictive value. Longitudinal studies are needed to determine whether these measures predict gait decline and represent modifiable therapeutic targets.

## Data Availability

The original contributions presented in the study are included in the article/Supplementary material, further inquiries can be directed to the corresponding author.

## References

[B1] Ai L. Li Z. Huang H. Huang C. Chen S. Zhou X. . (2025). Glymphatic system impairment in cerebral small vessel disease: associations with perivascular space volume and cognition. Front. Aging. Neurosci. 17:1680094. doi: 10.3389/fnagi.2025.1680094 41323655 PMC12657371

[B2] Alizadeh A. Van Opstal A. J. (2022). Dynamic control of eye-head gaze shifts by a spiking neural network model of the superior colliculus. Front. Comput. Neurosci. 16:1040646. doi: 10.3389/fncom.2022.1040646 36465967 PMC9714624

[B3] Berlot R. Rothwell J. C. Bhatia K. P. Kojovic M. (2021). Variability of movement disorders: the influence of sensation, action, cognition, and emotions. Mov. Disord. 36, 581–593. doi: 10.1002/mds.28415 33332680

[B4] Bower K. Thilarajah S. Pua Y. H. Williams G. Tan D. Mentiplay B. . (2019). Dynamic balance and instrumented gait variables are independent predictors of falls following stroke. J. Neuroeng. Rehabil. 16:3. doi: 10.1186/s12984-018-0478-4 30612584 PMC6322221

[B5] Cai M. Jacob M. A. Norris D. G. Duering M. de Leeuw F. E. Tuladhar A. M. (2021). Cognition mediates the relation between structural network efficiency and gait in small vessel disease. Neuroimage. Clin. 30:102667. doi: 10.1016/j.nicl.2021.102667 33887698 PMC8082689

[B6] Chong S. Wang S. Gao T. Yuan K. Han Y. Shi L. . (2025). Glymphatic function decline as a mediator of core memory-related brain structures atrophy in aging. J. Transl. Int. Med. 13, 65–77. doi: 10.1515/jtim-2025-0007 40115030 PMC11921812

[B7] Cicirelli G. Impedovo D. Dentamaro V. Marani R. Pirlo G. D'Orazio T. R. (2022). Human gait analysis in neurodegenerative diseases: a review. IEEE J. Biomed. Health. Inform. 26, 229–242. doi: 10.1109/JBHI.2021.3092875 34181559

[B8] Deravet N. Blohm G. de Xivry J. O. Lefevre P. (2018). Weighted integration of short-term memory and sensory signals in the oculomotor system. J. Vis. 18:16. doi: 10.1167/18.5.16 29904791

[B9] Elias-Mas A. Wang J. Y. Rodriguez-Revenga L. Kim K. Tassone F. Hessl D. . (2024). Enlarged perivascular spaces and their association with motor, cognition, MRI markers and cerebrovascular risk factors in male fragile X premutation carriers. J. Neurol. Sci. 461:123056. doi: 10.1016/j.jns.2024.123056 38772058 PMC12005344

[B10] Finsterwalder S. Wuehr M. Gesierich B. Dietze A. Konieczny M. J. Schmidt R. . (2019). Minor gait impairment despite white matter damage in pure small vessel disease. Ann. Clin. Transl. Neurol. 6, 2026–2036. doi: 10.1002/acn3.50891 31524338 PMC6801180

[B11] Fu X. Cai H. Quan S. Ren Z. Xu Y. Jia L. (2025). Immune cells in Alzheimer's disease: insights into pathogenesis and potential therapeutic targets. Med. Rev. 5, 179–202. doi: 10.1515/mr-2024-0064 40600183 PMC12207208

[B12] Gouveia-Freitas K. Bastos-Leite A. J. (2021). Perivascular spaces and brain waste clearance systems: relevance for neurodegenerative and cerebrovascular pathology. Neuroradiology 63, 1581–1597. doi: 10.1007/s00234-021-02718-7 34019111 PMC8460534

[B13] Hein Z. M. Che Mohd Nassir C. M. N. (2026a). Altered perivascular diffusion and elevated circulating cell-derived microparticles among individuals with enlarged perivascular spaces in subclinical cerebral small vessel disease. Exp. Neurol. 402:115783. doi: 10.1016/j.expneurol.2026.115783 41990907

[B14] Hein Z. M. Che Mohd Nassir C. M. N. (2026b). Fractal complexity of the circle of Willis links circulating microparticles to silent cerebral small vessel disease. Clin. Anat. doi: 10.1002/ca.70155. [Epub ahead of print]. 42365442

[B15] Hein Z. M. Che Mohd Nassir C. M. N. (2026c). Perivascular pathology, not macrovascular complexity, governs glymphatic-related dysfunction in preclinical cerebral small vessel disease. Sci. Rep. 16:4528. doi: 10.1038/s41598-026-36001-7 41530513 PMC12867987

[B16] Ineichen B. V. Okar S. V. Proulx S. T. Engelhardt B. Lassmann H. Reich D. S. (2022). Perivascular spaces and their role in neuroinflammation. Neuron 110, 3566–3581. doi: 10.1016/j.neuron.2022.10.024 36327898 PMC9905791

[B17] Jayakody O. Breslin M. Beare R. Siejka T. P. Gujjari S. Srikanth V. K. . (2021). The association between simple reaction time variability and gait variability: the Tasmanian study of cognition and gait. Gait Posture 89, 206–210. doi: 10.1016/j.gaitpost.2021.07.016 34340157

[B18] Kim S. L. Lee M. J. Lee M. S. (2014). Cognitive dysfunction associated with falls in progressive supranuclear palsy. Gait Posture 40, 605–609. doi: 10.1016/j.gaitpost.2014.07.005 25088758

[B19] Lin F. Yang B. Chen Y. Zhao W. Li B. Jia W. (2022). Enlarged perivascular spaces are linked to freezing of gait in Parkinson's disease. Front. Neurol. 13:985294. doi: 10.3389/fneur.2022.985294 36062021 PMC9437541

[B20] Loos C. M. McHutchison C. Cvoro V. Makin S. D. Staals J. Chappell F. . (2018). The relation between total cerebral small vessel disease burden and gait impairment in patients with minor stroke. Int. J. Stroke 13, 518–524. doi: 10.1177/1747493017730780 28906203

[B21] Osoba M. Y. Rao A. K. Agrawal S. K. Lalwani A. K. (2019). Balance and gait in the elderly: a contemporary review. Laryngoscope Investig. Otolaryngol. 4, 143–153. doi: 10.1002/lio2.252 30828632 PMC6383322

[B22] Pinkhardt E. H. Issa H. Gorges M. Jurgens R. Lule D. Heimrath J. . (2014). Do eye movement impairments in patients with small vessel cerebrovascular disease depend on lesion load or on cognitive deficits? A video-oculographic and MRI study. J. Neurol. 261, 791–803. doi: 10.1007/s00415-014-7275-1 24535136

[B23] Pinter D. Ritchie S. J. Doubal F. Gattringer T. Morris Z. Bastin M. E. . (2017). Impact of small vessel disease in the brain on gait and balance. Sci. Rep. 7:41637. doi: 10.1038/srep41637 28134332 PMC5278543

[B24] Sharma B. Wang M. McCreary C. R. Camicioli R. Smith E. E. (2023). Gait and falls in cerebral small vessel disease: a systematic review and meta-analysis. Age Ageing 52:afad011. doi: 10.1093/ageing/afad011 37000039 PMC10064981

[B25] Vassar R. L. Rose J. (2014). Motor systems and postural instability. Handb. Clin. Neurol. 125, 237–251. doi: 10.1016/B978-0-444-62619-6.00015-X 25307579

[B26] Wardlaw J. M. Benveniste H. Nedergaard M. Zlokovic B. V. Mestre H. Lee H. . (2020). Perivascular spaces in the brain: anatomy, physiology and pathology. Nat. Rev. Neurol. 16, 137–153. doi: 10.1038/s41582-020-0312-z 32094487

[B27] Wardlaw J. M. Smith C. Dichgans M. (2019). Small vessel disease: mechanisms and clinical implications. Lancet. Neurol. 18, 684–696. doi: 10.1016/S1474-4422(19)30079-1 31097385

[B28] Wilson J. Allcock L. Mc Ardle R. Taylor J. P. Rochester L. (2019). The neural correlates of discrete gait characteristics in ageing: a structured review. Neurosci. Biobehav. Rev. 100, 344–369. doi: 10.1016/j.neubiorev.2018.12.017 30552912 PMC6565843

[B29] Wu L. Wang Q. Zhao L. Jiang C. Y. Xu Q. Wu S. C. . (2020). Clinical and oculomotor correlates with freezing of gait in a Chinese cohort of Parkinson's disease patients. Front Aging Neurosci. 12:237. doi: 10.3389/fnagi.2020.00237 32903684 PMC7438737

[B30] Wu L. Wang Z. Zhou X. Kong Q. Zhang Y. Xu S. . (2024). Mismatch of MRI white matter hyperintensities and gait function in patients with cerebral small vessel disease. J. Magn. Reson. Imaging 60, 550–558. doi: 10.1002/jmri.29121 37921545

[B31] Xie H. Xia C. Zhao H. Xia Z. Zhang N. Huang Y. (2025). Variability, asymmetry and bilateral coordination of gait during single- and dual-task walking of patients with cerebral small vessel disease. Int. J. Neurosci. 135, 435–444. doi: 10.1080/00207454.2024.2309454 38294519

[B32] Yang S. Jiang J. Wang L. Zhao M. Li W. Duan Y. . (2025). Impact of enlarged perivascular spaces in the basal ganglia on gait in cerebral small vessel disease. Aging. Clin. Exp. Res. 37:138. doi: 10.1007/s40520-025-03045-0 40304928 PMC12043758

[B33] Yu L. Hu X. Li H. Zhao Y. (2022). Perivascular spaces, glymphatic system and MRI. Front. Neurol. 13:844938. doi: 10.3389/fneur.2022.844938 35592469 PMC9110928

[B34] Zhao P. Gu Y. Feng W. Xia X. Tian X. Du Y. . (2022). Gait disorders and magnetic resonance imaging characteristics in older adults with cerebral small vessel disease. J. Integr. Neurosci. 21:129. doi: 10.31083/j.jin2105129 36137968

[B35] Zhou X. Zhang C. Li L. Zhang Y. Zhang W. Yin W. . (2020). Altered brain function in cerebral small vessel disease patients with gait disorders: a resting-state functional MRI study. Front. Aging. Neurosci. 12:234. doi: 10.3389/fnagi.2020.00234 33110408 PMC7487416

