# Supplementary material for: Gait performance in cerebral small vessel disease: associations with cognitive and oculomotor function and exploratory mediation analyses
Source: Front Aging Neurosci. 2026 Sep 16;18:1935861. doi: 10.3389/fnagi.2026.1935861 (PMC13624108; doi:10.3389/fnagi.2026.1935861)
Supplement: Supplementary file 1 [file Data_Sheet_1.docx]

**Supplementary Methods**

**Gait assessment**

Gait performance was assessed using the Tinetti Balance and Gait Scale, the 3-m Timed Up and Go (TUG) test, and a wearable motion-analysis system.

The Tinetti Balance and Gait Scale comprises nine balance items and eight gait items, with maximum scores of 16 and 12 points, respectively. The total score is the sum of the balance and gait subscores, with lower scores indicating poorer performance.

Functional mobility was assessed using the 3-m TUG test. Participants wore their usual shoes and sat in a chair with armrests and a backrest, with their back against the backrest and their hands resting on the armrests. Participants who routinely used a walking aid held it during the test. A marker was placed 3 m in front of the chair. After the verbal command “Go,” participants stood up, walked 3 m at their usual pace, crossed the marker, turned around, walked back to the chair, turned again, and sat down. No physical assistance was provided. The examiner recorded the time from the participant’s back leaving the backrest to returning to a seated position, to the nearest 0.01 s. The test was performed three times, and the mean of the second and third trials was used for analysis.

Quantitative gait data were collected using the Ruiping V2.0 wearable motion and gait-analysis system (Hunan Gyenno Medical Technology Co., Ltd., Hunan, China). Ten inertial measurement unit sensors were placed on the chest, waist, bilateral wrists, bilateral thighs, bilateral ankles, and bilateral feet. During the assessment, participants performed five consecutive sit-to-stand repetitions and the 5-m TUG test. The system continuously recorded body movements, and raw motion signals were transmitted via Bluetooth to a host computer for processing. The test procedure was also recorded automatically by the system.

The gait parameters included stance-phase duration, swing-phase duration, cadence, gait velocity, step length, stride length, stride-length variability, turning time, and the total time required to complete five consecutive sit-to-stand repetitions. The stance phase was defined as the interval from heel strike to toe-off, and the swing phase as the interval from toe-off to the subsequent heel strike. Cadence was defined as the number of steps per minute. Step length was defined as the longitudinal distance between successive foot contacts of opposite feet, whereas stride length was defined as the distance between two successive contacts of the same foot. Stride-length variability represented the variation in stride length across consecutive gait cycles. Turning time was the duration of the turning phase during the 5-m TUG test. Sit-to-stand duration was the total time required to complete five consecutive sit-to-stand repetitions.

**Oculomotor assessment**

Oculomotor performance was assessed using the EyeKnow intelligent eye-tracking system (Beijing CAS-Ruiyi Information Technology Co., Ltd., Beijing, China). Eye movements were recorded using pupil-center/corneal-reflection tracking at a sampling rate of 90 Hz. Before testing, each participant underwent a five-point calibration procedure. The maximum calibration error radius was 2°. Recorded data were processed using the system’s built-in analysis module.

Each participant completed four standardized paradigms: overlap saccade, antisaccade, lateral gaze, and smooth pursuit.

In the overlap saccade task, a green target was initially presented at the center of the screen and then appeared at a randomly selected location 15° from the center along either the horizontal or vertical axis. Participants were instructed to shift their gaze to the target as quickly and accurately as possible. The outcome measures were saccade latency, accuracy, mean reaction time, and mean saccade velocity. Saccade latency was defined as the interval between target onset and saccade initiation. Accuracy was defined as the percentage of successful saccades among all trials. Mean reaction time was the average interval between target onset and completion of a correct saccade. Mean saccade velocity was calculated from the angular displacement and duration of the saccade.

In the antisaccade task, the same target configuration was used. Participants were instructed to suppress the reflexive saccade toward the target and instead direct their gaze to the mirror-symmetric location. The outcome measures were accuracy, mean reaction time, correction rate, and correction latency. Accuracy was defined as the percentage of direct and correct saccades to the mirror-symmetric location. Mean reaction time was the interval between target onset and completion of a correct antisaccade. Correction rate was defined as the proportion of trials in which an erroneous initial saccade was followed by a corrective saccade. Correction latency was the time required to complete the corrective saccade after its initiation.

In the lateral gaze task, participants fixated on a stationary green target, and fixation stability was assessed using total deviation duration, the number of gaze shifts exceeding 2°, cumulative deviation amplitude exceeding 4°, and fixation precision. Total deviation duration was the cumulative time during which gaze deviated by more than 4° from the fixation target. Cumulative deviation amplitude was the summed amplitude of deviations exceeding 4°. Fixation precision was defined as the proportion of task time during which gaze remained within the predefined fixation area.

In the smooth-pursuit task, participants followed a green target moving along a sinusoidal trajectory in the horizontal and vertical planes. The target amplitude was 20°, and the frequency was 0.2 Hz. Smooth-pursuit performance was quantified by the number of gaze deviations exceeding 4° and the cumulative amplitude of these deviations. A deviation was defined as an eye-position error greater than 4° relative to the moving target.
