# Supplementary material for: Gait performance in cerebral small vessel disease: associations with cognitive and oculomotor function and exploratory mediation analyses
Source: Front Aging Neurosci. 2026 Sep 16;18:1935861. doi: 10.3389/fnagi.2026.1935861 (PMC13624108; doi:10.3389/fnagi.2026.1935861)
Supplement: Supplementary file 2 [file Table_1.docx]

| **Supplementary Table 1. Overall and pairwise comparisons of clinical, imaging, gait, cognitive, and oculomotor measures among the NC, CSVD-NGD, and CSVD-GD groups** | | | | | | | | |
| --- | --- | --- | --- | --- | --- | --- | --- | --- |
| **Category** | **Variable** | **Overall *P*** | **NC vs CSVD-NGD** | | **NC vs CSVD-GD** | | **CSVD-NGD vs CSVD-GD** | |
|  |  |  | Raw *P* | Adjusted *P* | Raw *P* | Adjusted *P* | Raw *P* | Adjusted *P* |
| **Basic characteristics** | Age (years) | 0.100 | 0.169 | 0.282 | 0.061 | 0.122 | 0.338 | 0.462 |
|  | Women, n (%) | 0.512 | 0.328 | 0.462 | 0.308 | 0.462 | 0.932 | 0.932 |
|  | Body mass index (kg/m²) | 0.740 | 0.978 | 0.978 | 0.491 | 0.639 | 0.511 | 0.639 |
|  | Years of education | 0.904 | 0.651 | 0.744 | 0.739 | 0.813 | 0.926 | 0.932 |
| **Cerebrovascular risk profile** | Hypertension, n (%) | <0.001^***^ | 0.016^*^ | 0.032^*^ | <0.001^***^ | <0.001^***^ | 0.068 | 0.114 |
|  | History of stroke, n (%) | <0.001^***^ | <0.001^***^ | <0.001^***^ | <0.001^***^ | <0.001^***^ | 0.128 | 0.173 |
|  | Diabetes mellitus, n (%) | 0.909 | 1.000 | 1.000 | 0.695 | 0.773 | 0.695 | 0.773 |
|  | Atrial fibrillation, n (%) | 0.664 | 1.000 | 1.000 | 0.586 | 0.702 | 0.586 | 0.702 |
| **Neuroimaging** | Total CSVD load score | <0.001^***^ | <0.001^***^ | <0.001^***^ | <0.001^***^ | <0.001^***^ | 0.056 | 0.140 |
|  | Anatomical score for cerebral microbleeds | <0.001^***^ | 0.026^*^ | 0.043^*^ | <0.001^***^ | <0.001^***^ | 0.038^*^ | 0.095 |
| **Gait performance** | Timed up and go, s | <0.001^***^ | <0.001^***^ | <0.001^***^ | <0.001^***^ | <0.001^***^ | < 0.001^***^ | < 0.001^***^ |
|  | Gait speed, m/s | <0.001^***^ | <0.001^***^ | <0.001^***^ | <0.001^***^ | <0.001^***^ | < 0.001^***^ | < 0.001^***^ |
| **Cognition** | MMSE | <0.001^***^ | <0.001^***^ | <0.001^***^ | <0.001^***^ | <0.001^***^ | < 0.001^***^ | < 0.001^***^ |
|  | MoCA | <0.001^***^ | <0.001^***^ | <0.001^***^ | <0.001^***^ | <0.001^***^ | < 0.001^***^ | < 0.001^***^ |
|  | Trail making test A, s | <0.001^***^ | 0.019^*^ | 0.022^*^ | <0.001^***^ | <0.001^***^ | 0.003^**^ | 0.004^**^ |
|  | Trail making test B, s | <0.001^***^ | <0.001^***^ | <0.001^***^ | <0.001^***^ | <0.001^***^ | < 0.001^***^ | < 0.001^***^ |
|  | Clock drawing test score | <0.001^***^ | <0.001^***^ | <0.001^***^ | <0.001^***^ | <0.001^***^ | 0.004^**^ | 0.004^**^ |
|  | Judgment of line orientation score | <0.001^***^ | 0.002^**^ | 0.002^**^ | <0.001^***^ | <0.001^***^ | < 0.001^***^ | < 0.001^***^ |
| **Oculomotor function** | Lateral gaze deviation count (>2°) | 0.018^*^ | 0.532 | 0.620 | 0.033^*^ | 0.033^*^ | 0.005^**^ | 0.009^**^ |
|  | Overlap saccade latency (ms) | <0.001^***^ | 0.013^*^ | 0.024^*^ | <0.001^***^ | <0.001^***^ | 0.023^*^ | 0.049^*^ |
|  | Anti-saccade correct rate (%) | <0.001^***^ | 0.012^*^ | 0.024^*^ | <0.001^***^ | <0.001^***^ | 0.004^**^ | 0.009^**^ |
|  | Horizontal smooth pursuit deviation count (>4°) | <0.001^***^ | 0.029^*^ | 0.049^*^ | <0.001^***^ | <0.001^***^ | 0.002^**^ | 0.006^**^ |

Overall comparisons were performed using one-way ANOVA or the Kruskal–Wallis test, as appropriate, followed by multiplicity-adjusted pairwise comparisons. *P* values were adjusted using the Benjamini–Hochberg false discovery rate procedure. NC, non-case controls; CSVD, cerebral small vessel disease; CSVD-NGD, CSVD without gait disorder; CSVD-GD, CSVD with gait disorder. ^*^*P* < 0.05, ^**^*P* < 0.01, ^***^*P* < 0.001.
